# Ethanol Dehydrogenation over Copper-Silica Catalysts: From Sub-Nanometer Clusters to 15 nm Large Particles

**DOI:** 10.1021/acssuschemeng.2c06777

**Published:** 2023-07-20

**Authors:** Tomas Pokorny, Vit Vykoukal, Petr Machac, Zdenek Moravec, Nicola Scotti, Pavla Roupcova, Katerina Karaskova, Ales Styskalik

**Affiliations:** † Department of Chemistry, Masaryk University, Kotlarska 2, CZ-61137 Brno, Czech Republic; ‡ Consiglio Nazionale delle Ricerche, Istituto di Scienze e Tecnologie Chimiche “G. Natta”, Via Golgi 19, 20133 Milano, Italy; § Institute of Physics of Materials, Academy of Sciences of the Czech Republic, Zizkova 22, CZ-61662 Brno, Czech Republic; ∥ CEITEC Brno University of Technology, Purkynova 123, CZ-61200 Brno, Czech Republic; ⊥ Institute of Environmental Technology, CEET, VSB-TUO, 17. listopadu 2172/15, CZ-70800 Ostrava, Czech Republic

**Keywords:** ethanol dehydrogenation, copper, nanoparticles, acetaldehyde, sol−gel, dry impregnation

## Abstract

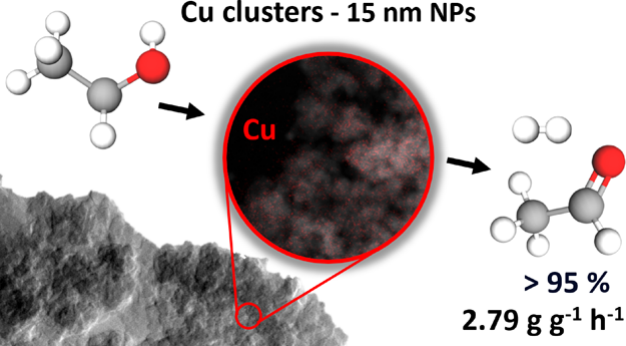

Non-oxidative ethanol dehydrogenation is a renewable source of acetaldehyde and hydrogen. The reaction is often catalyzed by supported copper catalysts with high selectivity. The activity and long-term stability depend on many factors, including particle size, choice of support, doping, etc. Herein, we present four different synthetic pathways to prepare Cu/SiO_2_ catalysts (∼2.5 wt % Cu) with varying copper distribution: hydrolytic sol–gel (sub-nanometer clusters), dry impregnation (*A̅* = 3.4 nm; σ = 0.9 nm and particles up to 32 nm), strong electrostatic adsorption (*A̅* = 3.1 nm; σ = 0.6 nm), and solvothermal hot injection followed by Cu particle deposition (*A̅* = 4.0 nm; σ = 0.8 nm). All materials were characterized by ICP-OES, XPS, N_2_ physisorption, STEM-EDS, XRD, RFC N_2_O, and H_2_-TPR and tested in ethanol dehydrogenation from 185 to 325 °C. The sample prepared by hydrolytic sol–gel exhibited high Cu dispersion and, accordingly, the highest catalytic activity. Its acetaldehyde productivity (2.79 g g^–1^ h^–1^ at 255 °C) outperforms most of the Cu-based catalysts reported in the literature, but it lacks stability and tends to deactivate over time. On the other hand, the sample prepared by simple and cost-effective dry impregnation, despite having Cu particles of various sizes, was still highly active (2.42 g g^–1^ h^–1^ acetaldehyde at 255 °C). Importantly, it was the most stable sample out of the studied materials. The characterization of the spent catalyst confirmed its exceptional properties: it showed the lowest extent of both coking and particle sintering.

## Abstract 2

Comparison of four preparation methods showed that the simple and cost-effective dry impregnation provides Cu/SiO_2_ material showing the most stable catalytic behavior in potential renewable acetaldehyde production despite having a broad particle size distribution.

## Introduction

Topical priorities in the chemical industry are developing sustainability, ecology, and the economy of production.^1−3^ Currently, the vast majority of acetaldehyde comes from the petrochemical industry^4−8^ and is produced by Wacker oxidation over a palladium catalyst.^9−11^ The ethylene precursor is produced in the steam cracking process. Acetaldehyde might be used as a butadiene precursor in the so-called Lebedev process.^12−15^ New sustainable catalytic pathways need to be developed to produce acetaldehyde and to overcome crude oil resource depletion.

Ethanol is an alternative source of both acetaldehyde and butadiene. Its dehydrogenation leads to acetaldehyde. This reaction also presents the first step of ethanol-to-butadiene transformation, known as the Lebedev or Ostromislensky process.^16,17^ It provides a possibility to substitute petroleum-based chemicals. Indeed, bioethanol is obtained from bio-sources in ever-increasing amounts, becoming an ideal platform molecule for the sustainable production of added-value chemicals. Its price decreases with the production increase.^18^

According to the literature, copper is a highly active and selective catalyst for the non-oxidative dehydrogenation of ethanol, but it suffers from deactivation by coking and particle sintering.^7,19−24^ The activity of various metals supported on carbon (Ce, Co, Cu, and Ni) was compared in a recent study. The copper-based catalyst was highly active and selective, reaching an ethanol conversion of 65.3% at 350 °C. In comparison, it ranged between 3.2 and 8% for Ce, Co, and Ni.^21^ The importance of the preparation method and size of the copper particles on silica (10 wt %) was shown in the study where ammonia evaporation (1.5–2.9 nm), deposition–precipitation (22.8 nm), and wet impregnation (83.5 nm) were compared. Surprisingly, the smallest nanoparticles were the most active and stable. Their outstanding thermal stability was attributed to the formation of the copper phyllosilicate phase.^19^ Various copper nanostructures (urchin-like, fiber-like, and nanorods) were prepared by microwave-assisted synthesis.^25^ The shape of particles impacted the catalytic activity, with urchin-like being the most active.^25^

A crucial role in the selectivity to acetaldehyde is played by the support. Up to now, we have presented catalysts supported on silica^19,25^ and carbon,^21^ which have little impact on acetaldehyde selectivity. For example, Zhang et al. have shown that copper-based catalysts supported on silica provided selectivity close to 100% at the temperature of 150 °C in ethanol dehydrogenation to acetaldehyde. The selectivity to acetaldehyde slightly decreased to ∼90% in samples with 20–50% of copper at higher temperatures (300 °C) due to the formation of ethyl acetate.^19^ Excellent acetaldehyde selectivity (∼98%) has also been reported by a research group led by Lu, when Cu was deposited on mesoporous carbon,^26^ N-doped carbon,^27^ and defect-rich boron nitride nanosheets.^28^ In contrary, the Cu/ZrO_2_ catalyst produced significant amounts of ethyl acetate at higher temperatures, with the selectivity to acetaldehyde reaching only 13–16%. The selectivity to ethyl acetate was 68–76% at 275 °C.^29^ A high selectivity to ethyl acetate was also observed by Fujita et al. in their study on Cu/ZnO catalysts.^30^ Finally, catalysts based on copper deposited on alumina and silica-alumina produce significant amounts of dehydration products (ethylene and diethyl ether) due to the acidity of the support.^19,31^

Stable Cu/SiO_2_ catalysts (500 h time-on-stream) were synthesized by the ammonia evaporation method, which provided highly dispersed particles, as already discussed. However, the authors mentioned that the number of available Cu sites in the stable catalysts exceeded the site requirement based on GHSV.^19^ Tu et al. reported the catalytic performance of 14 wt % Cu/SiO_2_ catalysts doped with Na, K, and Rb, prepared by dry impregnation. While the stability of Cu/SiO_2_, NaCu/SiO_2_, and RbCu/SiO_2_ catalysts was poor (loss of ∼50% activity in 4 h at 300 °C), the K-doped material exhibited more stable behavior.^32^ The stability can also be improved by a wise choice of the catalyst support. Li et al. showed that copper deposited on SiC or C/SiC exhibited a stable behavior during 8 h, while the Cu/SiO_2_ catalyst deactivated significantly faster.^33^ Similar, Cu deposited on N-doped carbon was stabilized by Cu–N interaction (according to XPS and DFT studies) and exhibited a stable catalytic behavior.^27^

The literature survey presented above shows the critical role of the particle size, choice of the support, and the preparation method in the catalytic activity, selectivity, and stability of copper-based catalysts in ethanol dehydrogenation. Herein, we report on a comparison of four different preparation methods for Cu NPs supported on porous SiO_2_: hydrolytic sol–gel, dry impregnation, strong electrostatic adsorption, and solvothermal hot injection. Particularly, we focused on the preparation of small Cu nanoparticles deposited on silica (from sub-nanometer clusters up to 15 nm) for the ethanol dehydrogenation to acetaldehyde. While the synthetic methods are well known and described in the literature,^34−38^ their application in ethanol dehydrogenation and comparison under identical catalytic conditions are new. Furthermore, we elucidated the structure of the Cu-based materials in detail, including the STEM-EDS analyses performed on all fresh calcined, fresh reduced, and spent catalysts. These experiments allowed us to estimate the particle size distribution, the reducibility of the copper phase, and the particle sintering during catalytic reaction. With that knowledge, we studied the catalytic activity, selectivity, and stability with time-on-stream and discussed the effect of particle size distribution in detail. The stability was tested at a relatively high temperature (325 °C) unlike other studies to achieve high acetaldehyde productivity and to find an ideal Cu-based catalyst suitable for butadiene production in the one-step Lebedev process.^5,39−42^ The same reason (i.e., the potential butadiene production) prompted us to work with silica support: it is thermally stable and allows for incorporation of other metals necessary for the next steps of the butadiene synthesis cascade.^7,15,41−43^

## Experimental Section

Cu(NO_3_)_2_·5/2H_2_O was used as a copper precursor (purchased from Merck). Cu was deposited by various methods (see below) on commercial silica Aerosil 300 from Evonik. One sample was prepared by the sol–gel technique from Si(OC_2_H_5_)_4_ (house stock; purified by vacuum distillation). Oleylamine (OLE) (technical purity, 70%) and 1-octadecene (ODE) (technical purity, 90%) were purchased from Merck, dried over sodium metal, vacuum-distilled, and stored under dry nitrogen.

### Preparation of Cu-Based Catalysts Supported on Porous SiO_2_

Cu/SiO_2_ catalysts were prepared by four methods: dry impregnation (DI), strong electrostatic adsorption (SEA),^44^ solvothermal hot-injection synthesis (SHI),^35−37^ and hydrolytic sol–gel (HSG).^38^ Cu was deposited on Aerosil 300 in the case of supported catalysts (DI, SEA, and SHI). All samples were calcined after preparation in an ambient atmosphere at 500 °C (10 °C min^–1^, 5 h). Nominal Cu loading was 2.5 wt % for all samples.

#### DI

Cu(NO_3_)_2_·5/2H_2_O (91.5 mg, 0.393 mmol) was dissolved in a minimal volume of water (10 cm^3^) needed to fill the pores of the Aerosil 300 support and form a thick paste. The solution of the precursor was mixed with the silica support (1.00 g). The sample was placed in an oven (70 °C). After the drying process, the sample was ground into a fine powder and calcined.

#### SEA

Cu(NO_3_)_2_·5/2H_2_O (183.0 mg, 0.787 mmol) was dissolved in water (0.6 dm^3^), and pH was set to 11.5 according to the literature^44^ by NH_3_ water solution. Aerosil 300 silica support (2.00 g) was added to the solution and left to react for 1 h. The sample was recovered by centrifugation, dried in an oven at 70 °C, and calcined.

#### SHI

Cu NPs were prepared by the solvothermal hot-injection (SHI) technique,^35−37^ characterized by TEM and UV–VIS (Figure S1), and then deposited on the porous support. SHI preparation was performed using Schlenk techniques under dry nitrogen/vacuum. Oleylamine (OLA; 90 cm^3^) and octadecene (ODE; 100 cm^3^) were added to the Schlenk vessel and heated up to 110 °C under vacuum for final purification. Cu(O_2_C_5_H_7_)_2_ (526.5 mg, 1.996 mmol) was dissolved in OLA (10 cm^3^) in a second Schlenk vessel. The mixture of OLA and ODE was heated to 230 °C under nitrogen, and the solution of the Cu precursor was injected into the hot mixture of OLA and ODE. The reaction mixture was kept at 230 °C for 10 min. Acetone (100 cm^3^) was added to the final Cu NPs solution after cooling down, and the reaction mixture was centrifuged. Nanoparticles were washed with an acetone–hexane mixture (ratio, 3:1) three times, dispersed in hexane (12 mL), and centrifuged repeatedly. Aerosil 300 support (1.00 g) was added to the colloidal solution of Cu NPs in hexane (copper amount in hexane analyzed by ICP-OES); the solution immediately decolored, while Aerosil became dark. Excess hexane was evaporated under a vacuum, and the resulting sample was calcined.

#### HSG

SiO_2_ containing copper was prepared by hydrolytic sol–gel from Si(OC_2_H_5_)_4_.^38^ Cu(NO_3_)_2_·5/2H_2_O was used as a copper precursor. All chemicals were mixed in the beaker (molar ratio TEOS:EtOH:H_2_O = 1:3.85:10.2), and the Cu precursor was calculated to form 2.5 wt % of Cu in the resulting Cu/SiO_2_ and was added into the reaction mixture. The reaction was left to hydrolyze and condense for 12 h in the ambient air. The excess concentrated NH_3_ water solution (5.0 cm^3^) was quickly added to the reaction mixture after 12 h to achieve the final gelation. The resulting blue gel was dried in an oven at 70 °C. The xerogel was then calcined in a tubular furnace.

### Characterization

An EMPYREAN instrument by the company PANalytical was used to measure powder X-ray diffraction. Samples were placed on a spinning sample bed. The Co lamp (λ = 1.78901 Å) was powered by 20 mA and 30 kV. A semiconductor detector was used in 1D mode.

Transmission electron microscopy (TEM) was carried out with an electron microscope FEI Tecnai F20 with an accelerating voltage of 200 kV and equipped with a 4 × 4k CCD camera. Samples were placed on a gold grid covered by a continuous carbon layer (12 nm).

For scanning transmission electron microscopy with electron dispersive X-ray spectroscopy (STEM-EDS), a device from the company FEI called Titan was used to obtain elemental maps. The excitation voltage was 300 V.

Particle size distribution evaluation was done by the graphic software ImageJ.^45^ Nanoparticles were measured crossway their longest side.

Cu dispersion was calculated from the average particle size estimated by STEM-EDS based on the cuboctahedron model. Assuming that the catalyst nanoparticles are cubooctahedral in shape with a face-centered cubic (fcc) structure, it is possible to calculate the number of surface atoms depending on the size of the nanocrystals. The total number of atoms for an fcc crystal can be calculated as follows:^46−48^
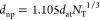
1where *d*_np_ is the average diameter of nanoparticles obtained by STEM-EDS, and *d*_at_ is the copper atom diameter (2.56 Å).

Equation 2 allows to calculate *m*, the edge length expressed in atoms.
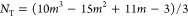
2

*N*_S_ (number of surface atoms) is given by eq 3:
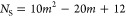
3

Finally, copper dispersion can be calculated as a ratio between *N*_S_ and *N*_B_:
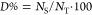
4

Thermogravimetry analysis (TGA) was measured by a Netzsch STA 449 C Jupiter. Samples were measured in Pt/Rh crucibles. The samples were heated in airflow (100 cm^3^ min^–1^), and the heating rate was 5 °C min^–1^ to 1000 °C.

Nitrogen porosimetry was measured using an Autosorb iQ3 (Quantachrome Instruments). Adsorption and desorption isotherms were measured at the temperature of −195.7 °C. Samples were degassed for at least 24 h at 200 °C. The specific surface area was determined by BET analysis from the measured isotherms in a relative pressure range from 0.05 to 0.30.

X-ray photoelectron spectroscopy (XPS) was measured on a Kratos Axis Supra equipped with a monochromatic source of X-ray with excitation Al Kα. A binding energy of 284.8 eV for C 1s was used for calibration.

Temperature programmed reduction was measured using a modified Pulse Chemisorb 2700 by the company Micromeritics. Samples were measured in a quartz reactor. Oxidation was carried out at 500 °C for 1 h with heating at 10 °C min^–1^ under O_2_ (40 cm^3^ min^–1^). The reduction was performed under 8% H_2_ in Ar with a heating program of 8 °C min^–1^ to 700 °C. The consumption of hydrogen was measured by a thermal conductivity detector.

Active copper surface area was determined by reactive frontal chromatography (RFC). A chemisorption analyzer AutoChemII 2920 (Micromeritics, USA) connected on-line with mass spectrometer HPR-20 EGA, Hiden Analytical, software MASsoft (Warrington, England) was used for measurement. Monitoring *m*/*z* = 28 (N_2_) and *m*/*z* = 44 (N_2_O) signals was used for calculation.

The catalyst amount of 0.7 g was used for RFC experiment. The description of individual steps of the whole RFC experiment is described as follows: (1) activation of catalysts at 350 °C for 1 h and cooling down to 50 °C in Ar flow (50 mL min^–1^); (2) reduction of catalysts by 10 mol % H_2_/Ar (30 mL min^–1^) while heating at 10 °C min^–1^ to 325 °C with subsequent isothermal reduction for 60 min at 325 °C; (3) hydrogen desorption in He (30 mL min^–1^) at 325 °C for 30 min; (4) cooling down in He (30 mL min^–1^) to 90 °C; (5) oxygen chemisorption in 3 mol % N_2_O/He (30 mL min^–1^) at 90 °C for 30 min; (6) reduction by 10 mol % H_2_/Ar (30 mL min^–1^) while heating at 10 °C min^–1^ to 250 °C with subsequent isothermal reduction for 30 min at 250 °C; (7) desorption of hydrogen in He (30 mL min^–1^) at 250 °C for 30 min; (8) cooling down in He (30 mL min^–1^) to 90 °C; (9) oxygen chemisorption in 3 mol % N_2_O/He (30 mL min^–1^) at 90 °C for 30 min.

The copper surface area (eqs 5 and 6) was calculated according to the method specified by Dvořák et al. and Chinchen et al.^49,50^ The number of adsorbed oxygen atoms in 1 m^2^ of copper surface area (0.697 × 10^19^; at O/m^2^ Cu) and the time obtained between changes of N_2_ and N_2_O signals (average value obtained during the first and second chemisorption steps) (steps 5 and 9) were used for calculation.
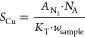
5where *S*_Cu_ is the copper surface area per gram of the sample (m^2^ g_sample_^–1^), *A*_N_2__ is the corrected amount of N_2_ released by decomposive N_2_O adsorption (0 °C, 101325 Pa) (mol), *N*_A_ is the Avogadro constant (6.022 × 10^23^ molecule per mol), *K*_T_ is the constant corresponding to the number of oxygen atoms adsorbed per m^2^ of copper at the temperature *T* (K) (*K*_363_ = 0.697 × 10^19^ atoms of O/m^2^ Cu^49^), and *w*_sample_ is the weight of the sample (g).
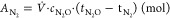
6where *V̇* is the flow rate of reaction gas (mL min^–1^), *c*_N_2_O_ is the N_2_O concentration in reaction gas (mol mL^–1^), and (*t*_N_2_O_ – *t*_N_2__) is the time distance between N_2_ and N_2_O signals during RFC N_2_O (min).

### Catalytic Ethanol Dehydrogenation to Acetaldehyde

A fixed-bed catalytic reactor connected to a gas chromatograph with a flame ionization detector was used for the catalytic reaction. The catalytic tests were performed at temperatures of 185, 220, 255, and 290 °C. One temperature step consisted of (i) a heating ramp (5 °C min^–1^) and stabilization at the set temperature (21 min) and (ii) a steady temperature state (60 min at 185 and 220 °C; 84 min at 255 and 290 °C). The analysis of the effluent gas was carried out by an HP 6890 Gas Chromatograph (five injections at 185 and 220 °C and seven injections at 255 and 290 °C) equipped with a flame ionization detector (FID) and a Thermo Scientific TG-BOND U column (30 m long, internal diameter of 0.32 mm, film thickness of 10 μm). The stability experiments were carried out for 14 h at 325 °C.^34^ Calcined catalysts (100 mg) with grain sizes between 0.2 and 0.4 mm were used for the catalytic reaction. All catalysts were adjusted to the same volume by glass beads (0.5–1 mm). The void space of the reactor was filled with silica beads. Before the reaction, the catalysts were pre-treated in situ by feeding hydrogen (5 vol % H_2_ in N_2_) for 2 h at 325 °C (CuO reduction). Nitrogen was used as carrier gas (50 cm^3^ min^–1^); ethanol was fed by a NE-300 syringe pump with WHSV of 4.73 h^–1^ (7.11 mol % of ethanol in N_2_). Pentane (5% molar concentration in ethanol feed) was used as an internal standard. The tests were carried out at atmospheric pressure.

## Results and Discussion

### Catalyst Preparation

Supported copper catalysts were prepared utilizing the commercial mesoporous silica Aerosil 300 as support. Three methods of copper deposition were used: (i) dry impregnation (DI), (ii) strong electrostatic adsorption (SEA),^44^ and (iii) solvothermal hot injection synthesis of NPs followed by their deposition on a silica surface (SHI).^35,36^ Finally, one sample was prepared by the hydrolytic sol–gel method for comparison (HSG).^38^

All samples were calcined after the copper deposition; thus, CuO particles were formed. The oxidized catalysts were characterized, reduced and characterized again, and finally reduced in situ before the catalytic reaction. The successful reduction of the copper species was confirmed by XPS, XRD, and H_2_-TPR (see below).

In almost all cases, the copper loading in the catalyst (Table 1) was slightly lower than the target (2.5 wt %) except for the SEA sample. During the preparation by the SEA method, the SiO_2_ support was exposed to NH_3_ and probably slightly dissolved due to high pH (pH = 11.5; yield loss observed), which led to a higher copper loading (2.91 wt %). Data gained by XPS spectroscopy showed a lower Cu surface concentration (0.40–0.85 wt %) for all samples (Table 1). The HSG sample exhibited similar bulk and surface Cu content to catalysts prepared by impregnation techniques.

**Table 1:** Experimental Cu Bulk and Surface Loadings in Cu/SiO_2_ Catalysts

preparation method	Cu loading [wt %]^a^	surface Cu concentration [wt %]^b^
DI	2.42	0.40
SEA	2.91	0.64
HSG	2.13	0.85
SHI	2.38	0.73

a Determined by ICP-OES.

b Estimated by XPS in fresh reduced samples.

### Porosity

**Figure 1:**
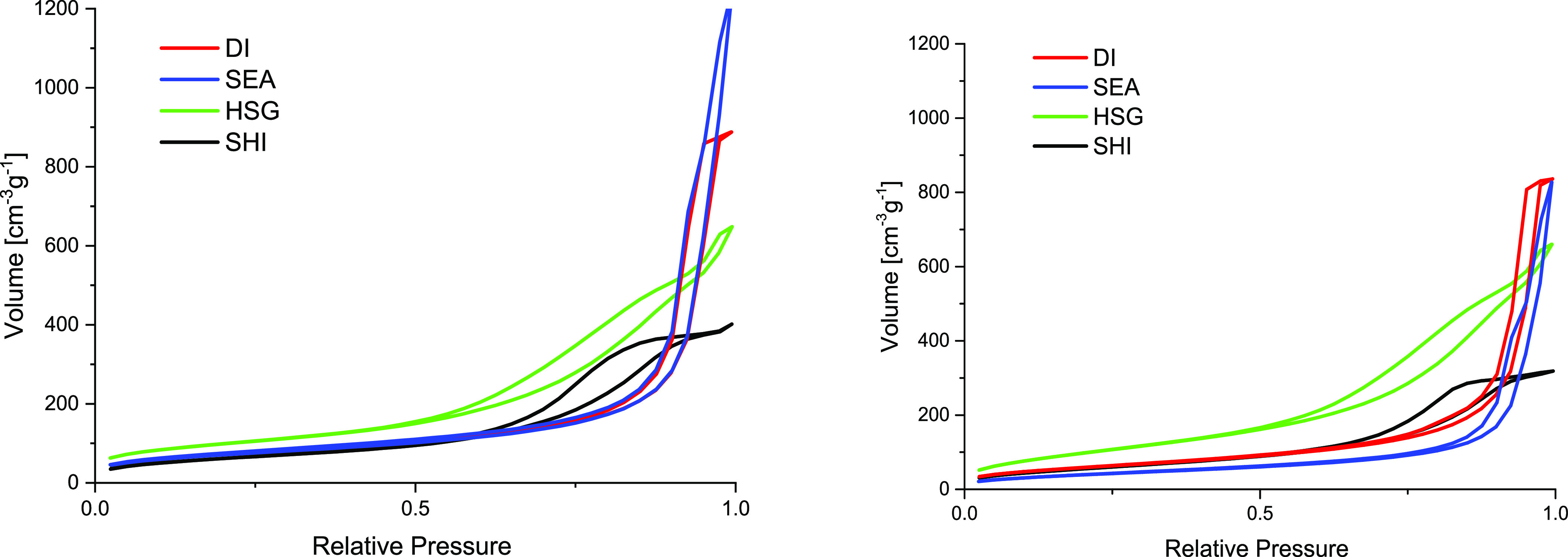
N_2_ adsorption–desorption isotherms of prepared Cu/SiO_2_ catalysts. Left: fresh reduced catalysts; right: spent catalysts.

Aerosil 300 (284 m^2^ g^–1^, 1.55 cm^3^ g^–1^, isotherm shown in Figure S2) was used for the preparation of DI, SEA, and SHI samples. Both specific surface area (SA_BET_) and total pore volume (*V*_total_) for all samples supported on Aerosil 300 decreased after the Cu NPs introduction (225–262 m^2^ g^–1^ and 0.59–1.44 cm^3^ g^–1^, respectively; Table 2). However, the N_2_ isotherms (Figure 1) are very similar to the isotherm of the parent material (Figure S2). They are represented by a steep N_2_ adsorbed volume increase at high partial pressures, indicating a high fraction of interparticle porosity.^51^ This is in line with the Aerosil 300 morphology (mixture of silica nano- and microparticles). Therefore, the DI, SEA, and SHI catalysts possess relatively high mean pore diameters (11–22 nm).

**Table 2:** Comparison of N_2_ Porosity before (Fresh Reduced) and after (Spent) Catalysis

	surface area [m^2^ g^–1^]			pore volume [cm^3^ g^–1^]			mean pore diameter [nm]		
preparation method	fresh	spent	surface area change [%]	fresh	spent	pore volume change [%]	fresh	spent	pore size change [%]
DI	262	218	**–17**	1.34	1.27	**–5**	20	23	**+15**
SEA	259	148	**–43**	1.44	0.86	**–40**	22	23	**+6**
HSG	355	381	**+7**	0.90	0.94	**+4**	10	9.8	**–2**
SHI	225	213	**–5**	0.59	0.48	**–19**	11	9.0	**–18**

The N_2_ isotherm of the HSG sample is different (close to the type IV isotherm typical for mesoporous materials). In agreement with the isotherm shape, the HSG sample displayed a higher SA_BET_ but a lower *V*_total_ (Table 2) with 10 nm average pore diameter. The isotherm shape suggests that the HSG sample is governed by intraparticle porosity contrary to the samples prepared from Aerosil 300, and the discussed differences originate in the sol–gel preparation.

### The Particle Size, CuO Reducibility, and Active Copper Surface Area

**Figure 2:**
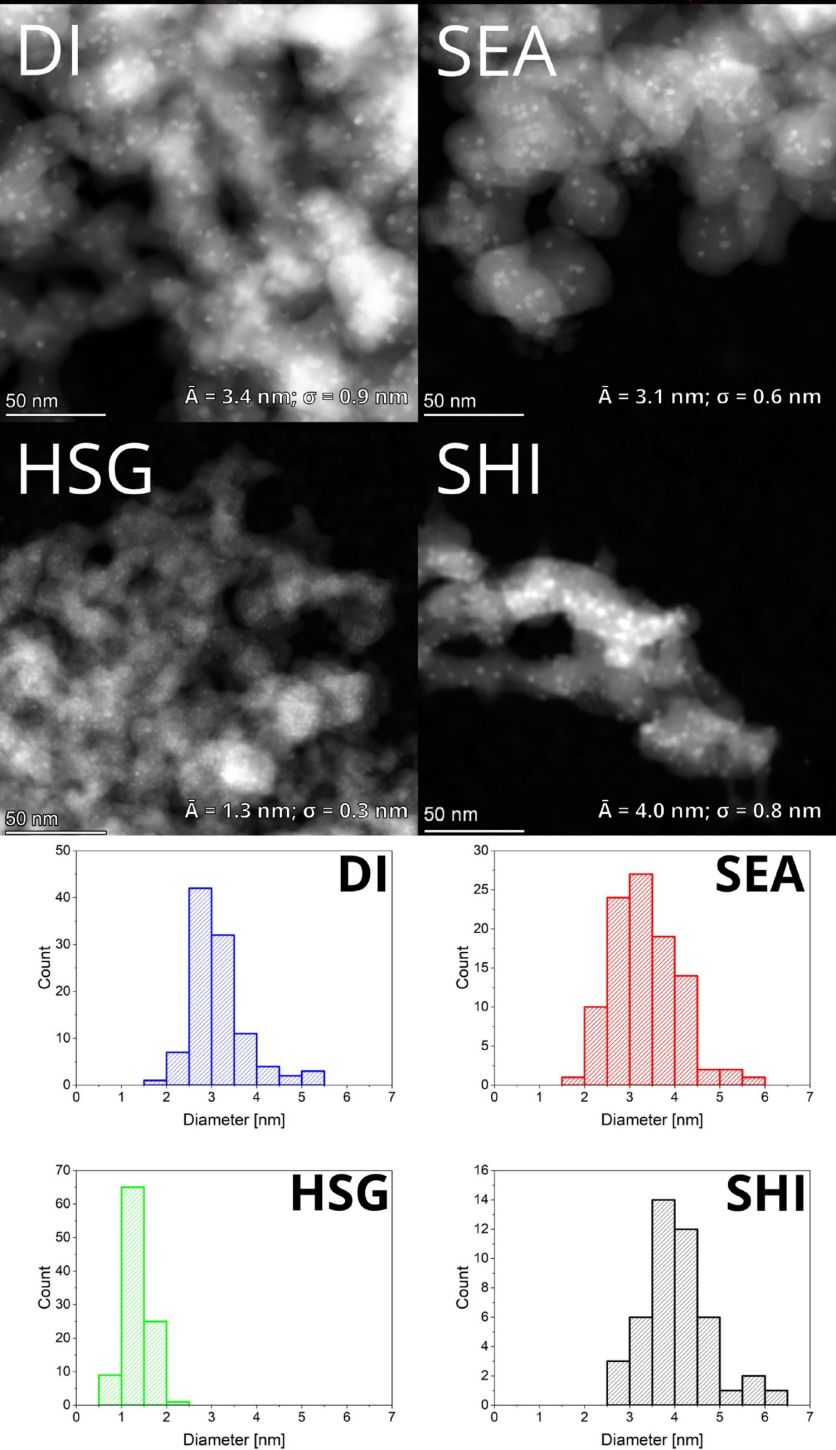
STEM-HAADF micrographs of the Cu nanoparticles in the samples and comparison of their particle size distributions using graphical analysis.

STEM with HAADF detector analysis (Figure 2) shows that the fresh reduced DI sample achieved particles with an average diameter of *A̅* = 3.4 nm and σ = 0.9 nm. SEA confirmed the advantage of using electrostatic forces between the negatively charged silica surface at pH = 11.5 and [Cu(NH_3_)_4_]^2+^ cations. Accordingly, nanoparticles in SEA were smaller with a narrower size distribution (*A̅* = 3.1 nm; σ = 0.6 nm), compared to DI, in agreement with the literature.^34^ For these two samples (DI and SEA), the size of the particles was very similar before (Figure S3) and after H_2_ treatment (Figure 2). SHI displayed large nanoparticles with the widest size distribution (*A̅* = 14.7 nm; σ = 3.2 nm) before their deposition on the silica surface (Figure S1). The large particles (∼15 nm) were still observed after deposition and calcination in air (Figure S3). However, the H_2_ treatment in this case led to significant changes in the particle size distribution: Particles with *A̅* = 4.0 nm and σ = 0.8 nm were observed in STEM micrographs. Interestingly, for HSG, a highly homogeneous Cu distribution was observed. Most of Cu was highly dispersed in sub-nanometer clusters before H_2_ treatment, and only a small part of Cu was present in particles (Figure S3). This fact comes from the synthesis, where Cu was incorporated within the sol–gel condensation step (one pot). The H_2_ treatment of HSG led to the formation of small and uniform particles (*A̅* = 1.3 nm and σ = 0.3 nm) homogeneously dispersed within the sample (Figure 2).

The data from STEM with HAADF detector analysis are summarized in Table 3, and they were used for the calculation of Cu NPs dispersion (*D*%) based on the assumption that the Cu particles adopt the shape of a cuboctahedron in the fcc structure.^46−48^ Indeed, the *D*% values follow the average particle sizes estimated by the graphical analysis of STEM micrographs. As some larger particles were observed by XRD in DI and SHI (see below), the *D*% values might be overestimated, especially for these two samples.

**Table 3:** Data Gained by Graphical Analyses of STEM Micrographs Regarding Particle Size, Particle Size Distribution, and Cu Dispersion^a^

sample	*A̅* [nm]	σ [nm]	*D*% [%]	active copper surface area [m^2^ g_sample_^–1^]
DI	3.4	0.9	33	1.6
SEA	3.1	0.6	36	2.4
HSG	1.3	0.3	68	0.9
SHI	4.0	0.8	28	1.4

a Active copper surface area determined by RFC.

The Cu dispersions based on the STEM analyses can be compared to the active copper surface areas determined by RFC using N_2_O as a selective oxidant for surface Cu atoms. The temperature used for Cu reduction was kept consistent with the pre-catalysis reduction (325 °C). The results obtained for samples prepared using impregnation techniques (DI, SEA, and SHI) correlated with the data obtained from STEM analyses. SEA exhibited the largest amount of surface copper area (2.4 m^2^ g^–1^) with nanoparticles measuring 3.1 nm in size. As the size of the reduced copper nanoparticles increased, the active surface copper area decreased (DI: 1.6 m^2^ g^–1^ with 3.4 nm nanoparticles, followed by SHI: 1.4 m^2^ g^–1^ with 4.0 nm nanoparticles; see Table 3). However, the HSG sample stood out from this trend. Despite having the smallest particles according to STEM analyses (1.3 nm), it achieved the smallest active Cu surface area (0.9 m^2^ g^–1^). This result is likely due to incomplete reduction of Cu species before RFC N_2_O treatment: (i) Significant H_2_ consumption was observed for HSG in TPR measurements within the temperature range of 400 to 700 °C (see below), and (ii) the reduction of HSG at higher temperatures (500 and 600 °C) before RFC N_2_O treatment led to a significant increase in measured active copper surface area (1.5 and 1.9 m^2^ g^–1^, respectively). Earlier studies have shown that Cu species highly dispersed in silica are only reduced at high temperatures.^52^ Therefore, the RFC method is not suitable for evaluation of copper surface area with highly dispersed species reducible only at high temperature range as was already stated by several authors.^52,53^

**Figure 3:**
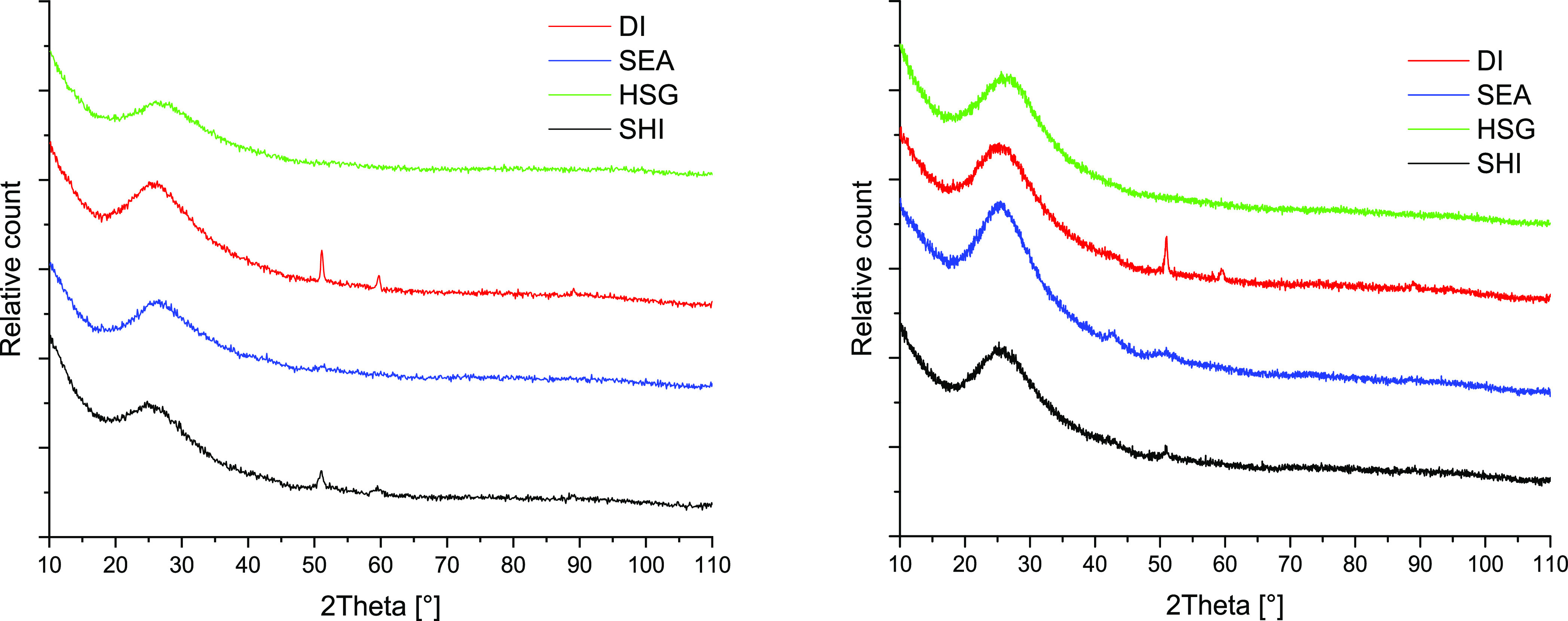
XRD diffractograms of Cu/SiO_2_ catalysts. Left: fresh reduced catalysts. Right: spent catalysts. Diffractions of metallic copper were observed (98-062-7113).

The powder X-ray diffraction data of fresh reduced samples (Figure 3, left) are in good agreement with STEM-EDS results for SEA and HSG: No diffraction maxima were observed for SEA and HSG due to the presence of very small NPs (smaller than 5 nm according to STEM-EDS). SHI exhibited diffractions of Cu due to the presence of larger nanoparticles. The average coherent domain size estimated by the Debye–Scherrer equation (∼13 nm) is in good agreement with TEM data before particle deposition (Figure S1) and with STEM-EDS micrographs after calcination in air (Figure S3) but differs from the average particle size estimated from STEM-EDS micrographs after H_2_ treatment (Figure 2). The DI sample showed narrower diffractions indicating the presence of some larger Cu crystallites (∼32 nm) in addition to small NPs observed in STEM-EDS micrographs. The larger particles (up to 8 nm) were observed in DI only when doing survey STEM-EDS analyses (Figure S4). The discrepancy between STEM-EDS and XRD analyses for DI and SHI originates in the limitation of the STEM-EDS method, which allows observing only a small part of the sample. Finally, the comparison of XRD patterns of fresh oxidized (Figure S5) and fresh reduced samples (Figure 3, left) shows a successful reduction of CuO species to Cu particles. The width of diffractions remains similar for both DI and SHI, indicating no dramatic changes in crystallite sizes.

**Figure 4:**
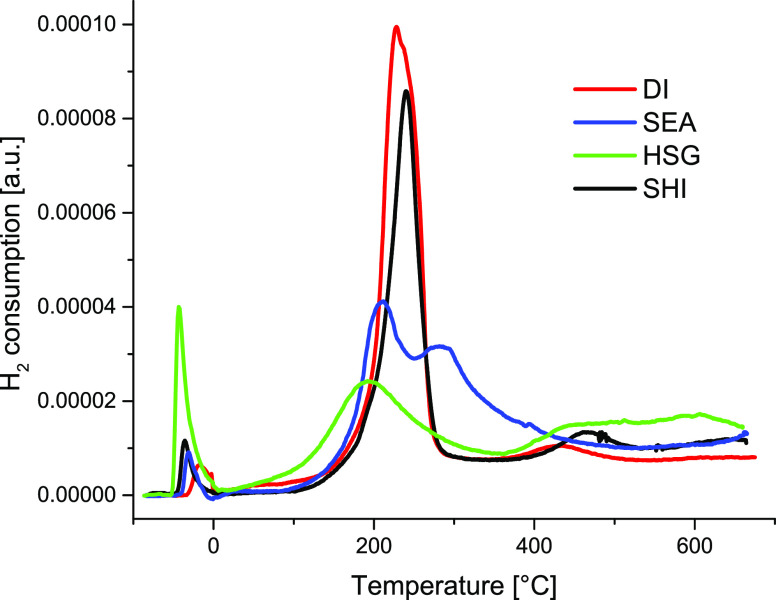
H_2_-TPR patterns for prepared Cu/SiO_2_ catalysts.

H_2_-TPR analysis was performed for all samples from −50 to 700 °C (Figure 4). The position and shape of the main peak could be related to the size of nanoparticles: the lower the reduction temperature, the smaller the particles.^54^ The maxima of the main peak lies between 191 and 248 °C with an on-set temperature of 100 °C (except for the HSG sample, see below). A sharp main peak at 241 °C in SHI evidenced the presence of copper particles with uniform dispersion. The DI catalyst exhibited the main peak at 228 °C. The lower temperature, in comparison to the SHI sample, fits with the presence of smaller nanoparticles observed by STEM-EDS. Nevertheless, the peak is broader, indicating a broad distribution of particle sizes. This observation can be related to the presence of some larger particles in agreement with XRD analyses. The H_2_-TPR result for the SEA sample shows a sharp main peak at 211 °C that can be ascribed to the reduction of well-dispersed and uniform nanoparticles, again, in agreement with STEM-EDS measurement. A second broad peak occurs in the SEA sample at 284 °C, representing either reduction of bulk CuO^29^ or different Cu/SiO_2_ species originating in the preparation method applying high pH (possibly copper phyllosilicate phase).^19^ As no bulk CuO was observed by XRD, the second explanation seems to be more probable. The HSG catalyst exhibited the main peak at the lowest temperature (191 °C). The on-set temperature of 31 °C only suggests the presence of highly active copper nanoclusters and well-dispersed copper atoms, in agreement with STEM-EDS analyses. The uptake of hydrogen occurred up to 700 °C, representing a reduction of Cu^2+^ in a stronger interaction with SiO_2_ (probably highly dispersed Cu species in silica).^52,53^ Some minor peaks were observed in this region also for DI (429 °C) and SHI (468 °C) samples. H_2_-TPR at low temperatures (from −50 to 0 °C) shows a minor peak for all samples (HSG: −42.2 °C; SEA: −32.7 °C; DI: −15.6 °C; SHI: −34.6 °C). According to Wang and Yeh, this low-temperature event can be related to surface reduction.^55^ Thus, the HSG catalyst, with mostly high Cu dispersion and the smallest particles, exhibited the most intense peak in this temperature range.

**Figure 5:**
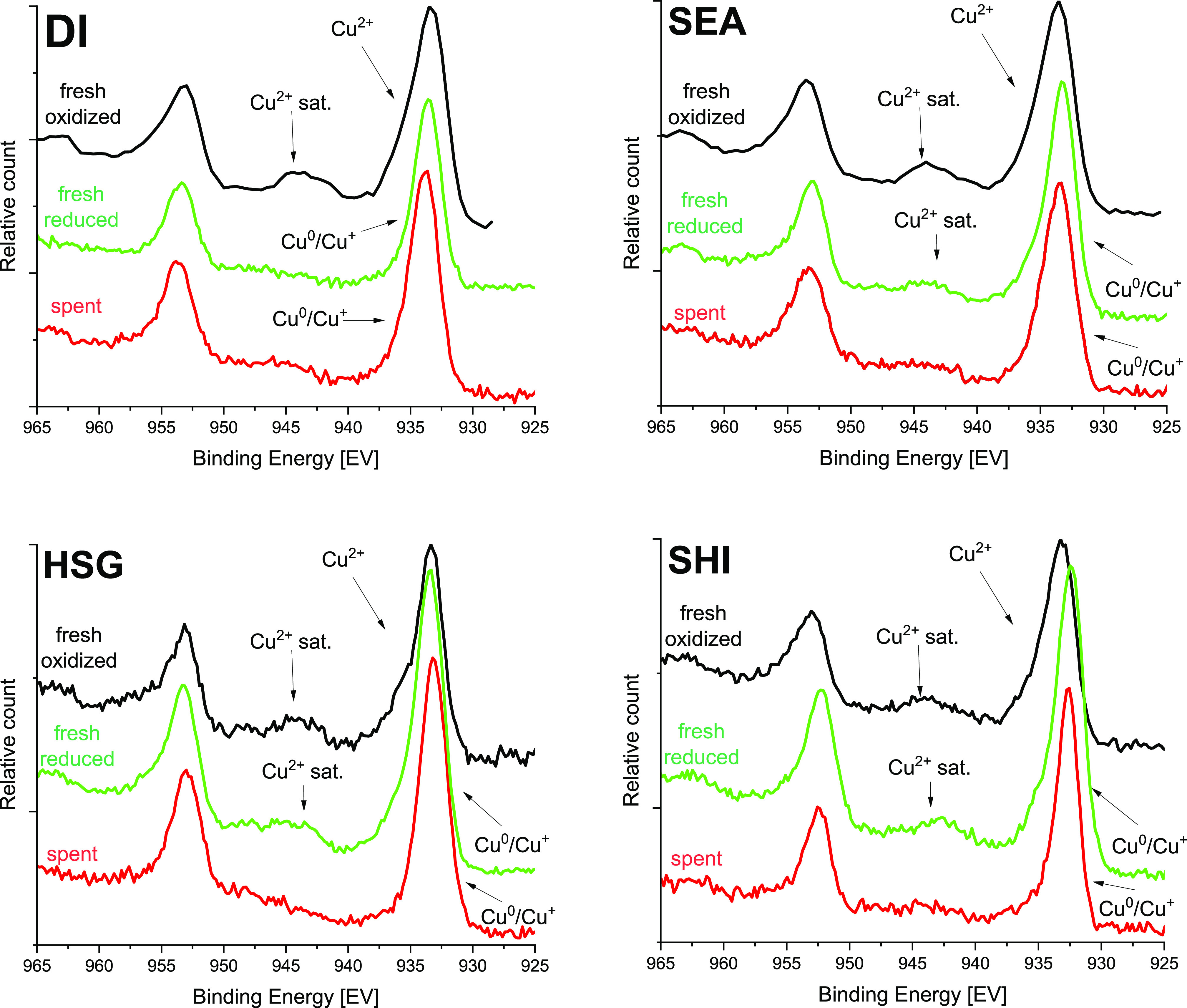
Cu 2p XPS spectra of fresh oxidized (black), fresh reduced (green), and spent catalysts (red).

X-ray photoelectron spectroscopy (XPS) was applied to follow the copper oxidation states (Figure 5). The presence of a satellite peak at ∼943 eV in the Cu 2p XPS spectra proves that Cu^2+^ was prevalent in all fresh oxidized samples.^27,28^ The satellite peak disappeared for DI after H_2_ treatment (325 °C, 2 h), indicating its successful reduction to Cu^0^ and probably Cu^+^ species.^27,28^ This result is in good agreement with the H_2_-TPR method, where the main H_2_ consumption appeared at 228 °C, and with XRD, where diffractions of metallic Cu were observed. The Cu LMM spectra were collected to distinguish the Cu^0^ and Cu^+^ oxidation states in DI (Figure S6).^27,28^ Unfortunately, the surface Cu concentration is low and the Cu LMM signals are buried in the base of the intense O 1s peak. Therefore, it is not possible to draw any conclusion. The presence of both Cu^0^ and Cu^+^ species can be expected on the catalyst surface, similar to other reports.^27,28^ In other catalysts (SEA, HSG, and SHI), the satellite peak in the Cu 2p XPS spectra did not disappear completely after the H_2_ treatment, suggesting the presence of some Cu^2+^ species (Figure 5). However, the samples were exposed to air for a short time necessary for their manipulation (e.g., preparation for XPS analysis) and, therefore, samples could have been re-oxidized during that period. Finally, all samples show a complete disappearance of the satellite peak characteristic for the Cu^2+^ oxidation state after the catalytic reaction.

### Catalytic Ethanol Dehydrogenation to Acetaldehyde

**Figure 6:**
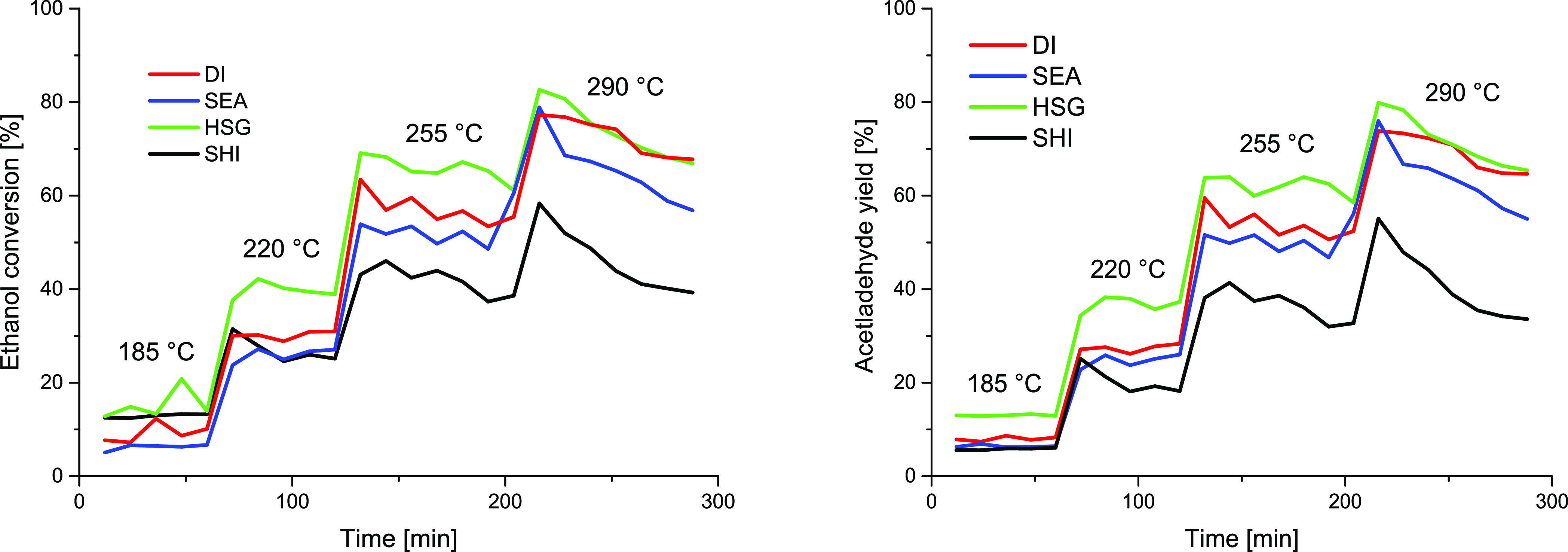
Comparison of the catalytic performance of the Cu/SiO_2_ catalysts prepared by different synthetic routes in ethanol-to-acetaldehyde catalytic reaction. Left: ethanol conversion. Right: acetaldehyde yield.

The results of the ethanol dehydration reaction are summarized in Figure 6. For all measurements, the carbon balance ranged between 95 and 100%. The main product of ethanol dehydrogenation in all prepared catalysts was acetaldehyde; the minor product was ethylene (up to 5%). Ethyl acetate was not observed in the reaction products, similar to other reports studying ethanol dehydrogenation over Cu NPs supported on silica.^56^ Details on ethanol conversion, stability test, and acetaldehyde selectivity are reported in Tables S1–S3. It is worth noting that the high acetaldehyde selectivity is maintained even at high temperatures. Acetaldehyde selectivity for DI, SEA, and HSG at 290 °C was nearly 100%, while SHI was the only sample to exhibit somewhat lower selectivity during the whole catalytic experiment. For these reasons, the acetaldehyde yield (Figure 6, right) closely followed the ethanol conversion plots (Figure 6, left) and will not be discussed in more detail.

The ethanol conversion increased with increasing temperature (Figure 6, left). The HSG catalyst with high Cu dispersion outperformed the others at each temperature in catalytic performance. The ethanol conversion at 255 °C (data not affected by sample instability) decreased in the order HSG > DI > SEA > SHI, with the catalyst bearing the largest particles (SHI) being the least active one. Interestingly, the DI catalyst with the broad particle size distribution (from *A̅* = 3.4 nm with σ = 0.9 nm according to STEM-EDS and up to ∼32 nm crystallite size according to XRD) outperformed at higher temperature (290 °C) the SEA sample with very uniform and smaller particles on its surface. Porosity was very similar for these two catalysts (both supported on Aerosil 300, similar SA_BET_ and *V*_total_ values) and thus did not play a significant role in the catalytic activity difference. The discrepancy can be explained by the H_2_-TPR measurement, which revealed two peaks at 211 and 284 °C for SEA, indicating poor reducibility of a significant portion of the introduced copper. Noteworthily, HSG also showed poor reducibility of some Cu species, but the highly dispersed Cu species in HSG exhibited the best catalytic activity among the systems studied.

In addition, the acetaldehyde productivity of our samples was compared to the literature reports (Table 4). Interestingly, the HSG sample reached the second highest acetaldehyde productivity from the listed catalysts (Table 4). Surprisingly, this catalyst contains the lowest copper loading out of the chosen materials. The fourth sample in acetaldehyde productivity is DI, which produced 2.42 g g^–1^ h^–1^ at 255 °C. The best reported catalyst (5 wt % Cu on defect-rich boron nitride nanosheets) outperforms all listed catalysts with a stable productivity of 6.72 g g^–1^ h^–1^.^28^ The best-reported catalyst deposited on silica (Cu/SiO_2_-AE)^23^ with a copper loading of 2.7 wt % produced an amount of acetaldehyde (2.44 g g^–1^ h^–1^) comparable to DI and lower than HSG. This material exhibited high Cu dispersion with nanoparticles up to 2.5 nm. The other catalysts with higher Cu content (5–25 wt %) showed much poorer catalytic performance (Table 4).

**Table 4:** Comparison of Acetaldehyde Productivity with Literature Data

sample	Cu [wt %]	WHSV [h^–1^]	temperature [°C]	ethanol conversion [%]	acetaldehyde selectivity [%]	acetaldehyde productivity [g g^–1^ h^–1^]
HSG (this work)	2.13	4.73	255	65	>95	2.79
DI (this work)	2.42	4.73	255	57	94	2.42
Cu/SiO_2_^23^	25	2.37	260	45	94	0.96
Cu/SiO_2_-AE^19^	2.7	3.16	250	85	>95	2.44
Cu/Beta zeolite^20^	5	1	250	65	89	0.55
5Cu/ZrO_2_^29^	5	3.16	250	64	17	0.33
Cu/SiO_2_/SiC^33^	5	2.4	260	70	90	1.45
Cu/SiO_2_^57^	8.4	2.4	260	85	80	1.56
Cu/C^26^	10	2.4	260	17	99	0.40
Cu/N-doped C^27^	10	2.4	260	83	94	1.87
Cu/BNS^28^	5	9.8	260	70	98	6.72

The catalyst stability with time-on-stream is another important parameter. HSG, SEA, and DI provided stable behavior at temperatures of 185, 220, and 255 °C for at least 1 h (Figure 6). The SHI catalyst was unstable already at 220 °C with a steep decrease in catalytic activity. All catalysts deteriorated their activity at 290 °C. The DI and HSG catalysts lost during a 1.5 h measurement 10–15% of catalytic activity. Even higher deactivation was recorded for the SEA and SHI catalysts, with a drop of more than 20% during this time (Figure 6).

**Figure 7:**
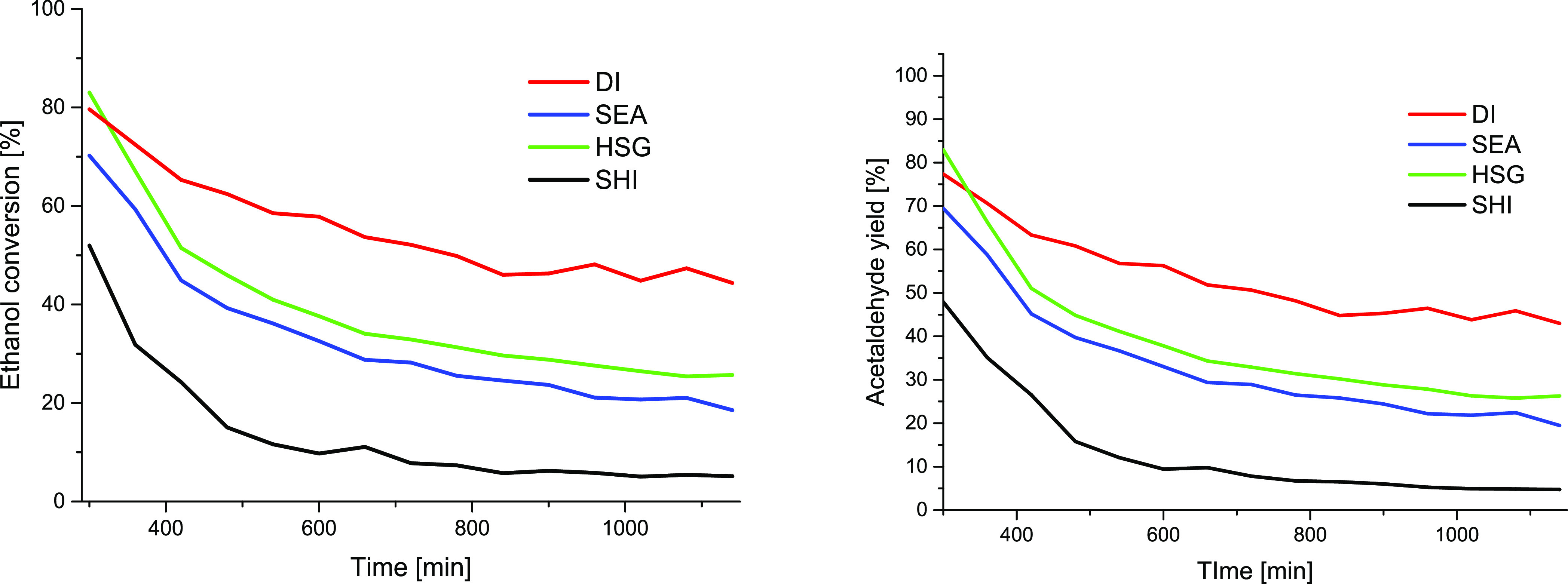
Catalytic stability of the Cu/SiO_2_ catalyst during ethanol dehydrogenation at 325 °C. Left: ethanol conversion. Right: acetaldehyde yield.

The longer stability tests (additional 14 h, after the light-off analyses) were performed at 325 °C. Such a high temperature was chosen to possibly produce the acetaldehyde for the Ostromislensky or Lebedev process (ethanol-to-butadiene reaction), which is usually performed at temperatures between 300 and 400 °C.^5,7,41^ The data in Figure 7 and Table S2 confirm that the Cu-based catalysts are unstable at high temperatures, similar to another report.^19^ The carbon balance and acetaldehyde selectivity at this high temperature were almost 100% during the whole catalytic test duration (Figure 7, right). All samples suffered from massive deactivation in the first 200 min of the stability test (i.e., time of 300–500 min), especially SHI. SEA and HSG behave similarly, probably due to the coking and sintering of very small particles, which are unstable under the given reaction conditions (see Spent Catalyst Characterization). No difference originating in different textural properties in SEA (interparticle porosity) and HSG (intraparticle porosity) has been recorded. Interestingly, larger nanoparticles with a broader particle size distribution found in the DI sample (*A̅* = 3.4 nm; σ = 0.9 nm by STEM-EDS; ∼32 nm by XRD) resulted in higher stability. After more than 14 h, the DI catalyst still shows an ethanol conversion of 44%. In contrary, the SHI sample with ca. 13 nm NPs (by XRD) exhibited the least stable behavior with ethanol conversion lower than 5% after 12 h (Table S2).

### Spent Catalyst Characterization

The samples were characterized before (fresh catalysts) and after catalysis (spent catalysts) to determine the mechanism of catalysts’ deactivation. The mass increase was measured by thermogravimetry, and the surface carbon content was followed by XPS (Table 5) to evaluate coking. In all catalysts, the carbon content increased during the catalytic reaction. According to both measurements (TGA and XPS), the smallest amount of carbon is formed on the catalyst’s surface of the DI catalyst (+1.75 and 0.88% according to TGA and XPS, respectively). Noteworthily, this sample exhibited the best stability during the catalytic test at 325 °C. Other catalysts showed an increase in mass loss by 1.79–3.50% (TGA) and surface carbon concentration by 2.83–3.60% (XPS). All of those catalysts were less stable during the stability measurements.

**Table 5:** Estimation of Coking by Thermogravimetry and XPS

	mass change (TGA) [%]			surface carbon content (XPS) [wt %]		
preparation method	fresh	spent	coking by TGA [%]	fresh	spent	carbon content increase (XPS) [wt %]
DI	0.91	2.66	**+1.75**	4.57	5.45	**+0.88**
SEA	3.06	4.85	**+1.79**	2.68	6.28	**+3.60**
HSG	3.42	6.89	**+3.47**	3.79	6.61	**+2.83**
SHI	0.55	4.05	**+3.50**	2.58	6.47	**+3.47**

These results suggest that one of the deactivation processes involves coke formation, which might cover the active copper sites and fill and clog the pores. The evidence of possible pore blockage was observed by N_2_ porosimetry performed on spent samples. A decrease in SA_BET_ (−5 to −17%) and *V*_total_ (−5 to −40%) indicated the negative effect of coking on the porosity of the catalysts (Table 2 and Figure 1, right). DI and HSG showed stable porosity properties. On the contrary, SEA and SHI were strongly affected (−40 and −19% *V*_total_ for SEA and SHI, respectively).

**Figure 8:**
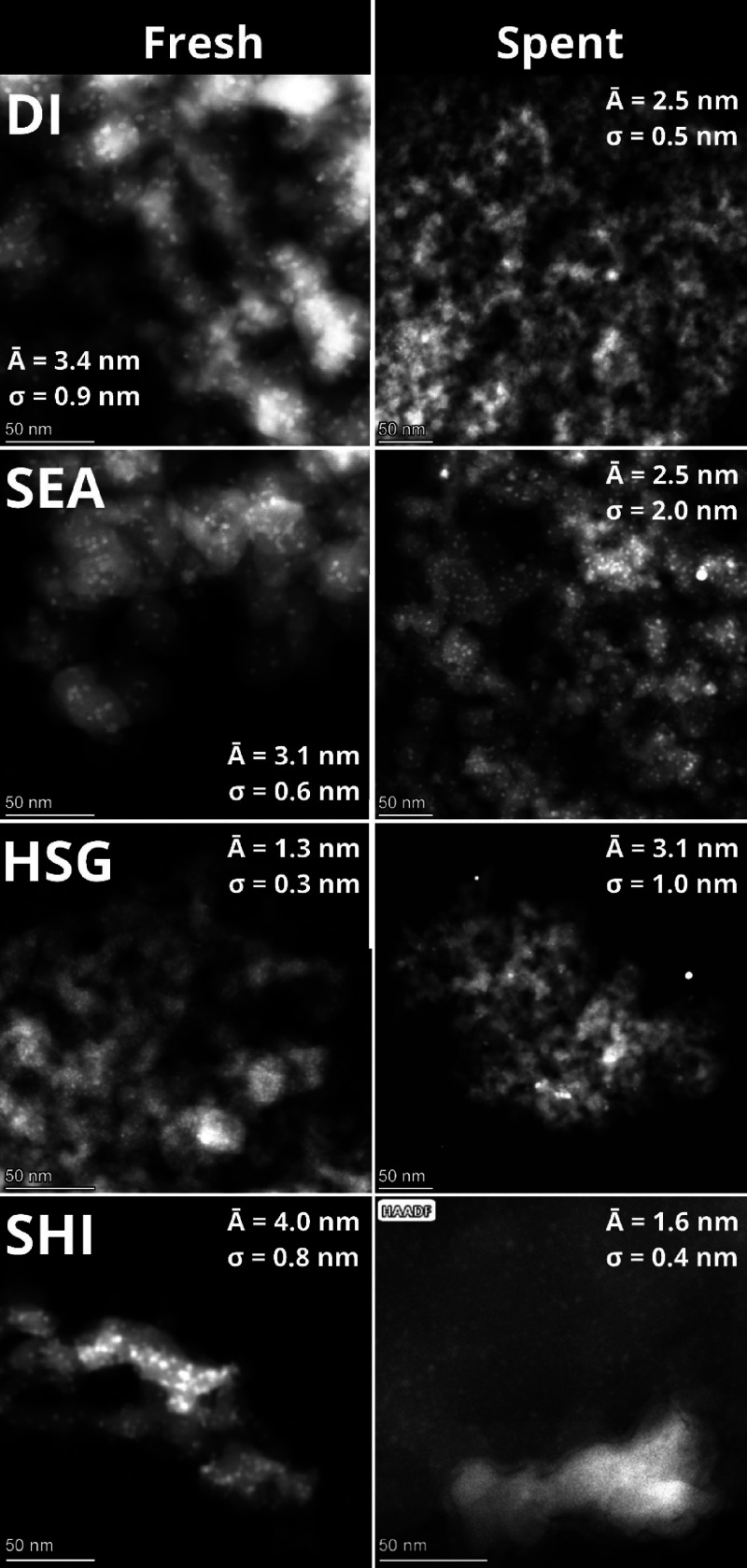
Sintering of the Cu-based catalyst during the catalysis observed by STEM with a HAADF detector. Left: fresh reduced samples. Right: spent catalysts. Several bright spots in the micrographs of SEA and HSG catalysts are Au nanoparticles that contaminated samples during their preparation for STEM-EDS analysis.

**Figure 9:**
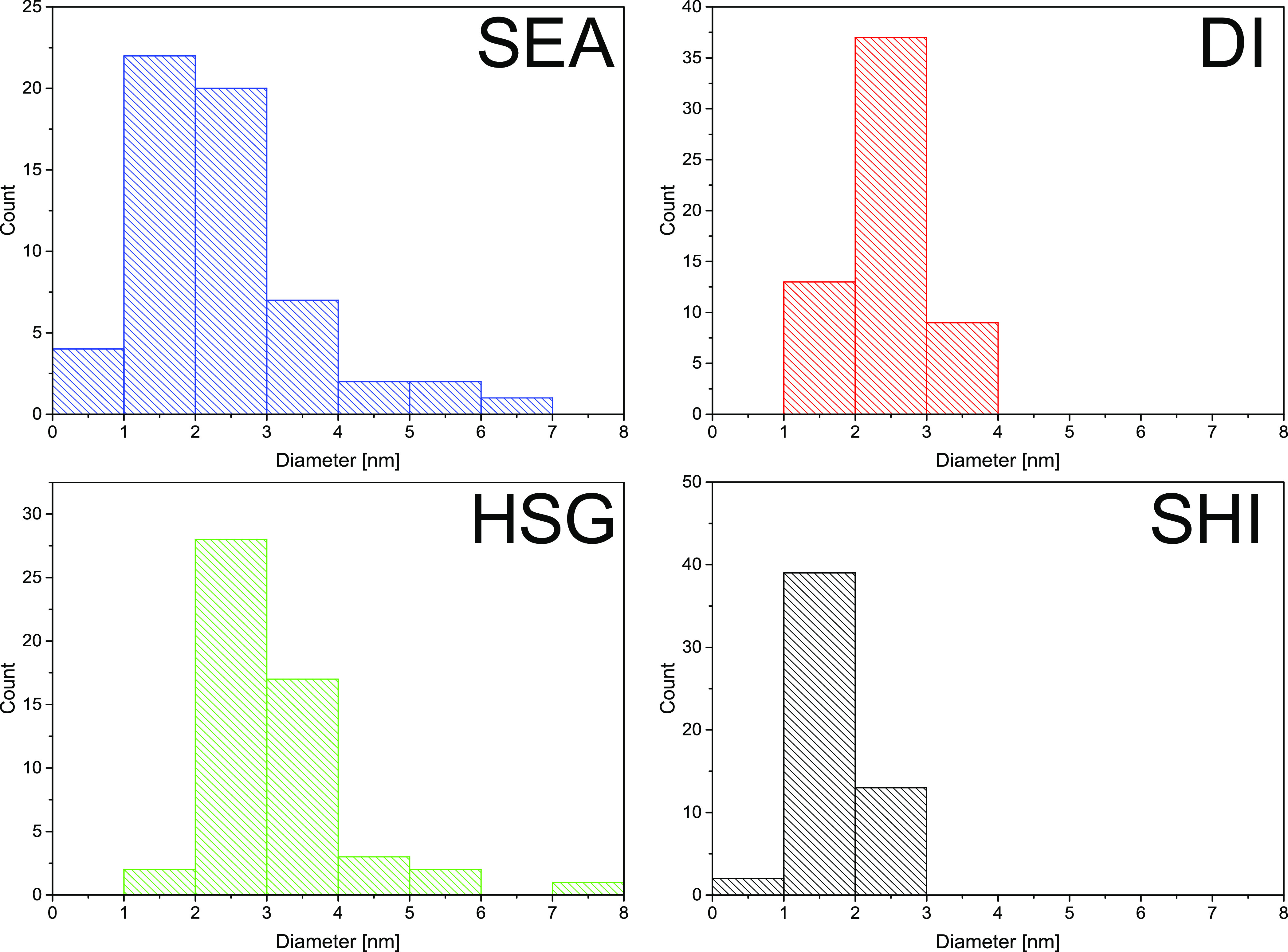
Particle size distribution histograms of copper nanoparticles in the spent catalysts from STEM micrographs with a HAADF detector.

Another common phenomenon that causes deactivation is the sintering of nanoparticles. Thus, the spent catalysts were analyzed again by STEM with a HAADF detector (Figure 8) and STEM-EDS (Figure S7). Interestingly, DI did not significantly sinter during the catalytic reaction; the micrographs of the spent DI catalyst show even smaller particle size with narrower distribution than before catalysis. This is due to the limitation of the STEM-EDS method, which allows observing only a small part of the sample. Still, this result is in good agreement with the observed stability of the DI catalyst. The most significant particle size growth during the catalysis occurred in the case of SEA and HSG (Figures 8 and 9). In the case of the SEA catalyst, the particle size distribution mainly broadened (*A̅* = 2.5 nm; σ = 2.0 nm). In the case of the HSG sample, the average particle size increased from 1.3 to 3.1 nm and the particle size distribution characterized by σ broadened (from 0.3 to 1.0 nm). Finally, only small particles (∼1.6 nm) were observed in the micrographs of the SHI sample after catalysis, indicating enormous Cu migration within the silica support surface.

Cu NPs sintering and crystallization were also followed by the XRD measurements (Figure 3, right). Diffractions of metallic copper were observed in agreement with XPS data collected on spent catalysts. The XRD and XPS data showing that the catalysts are mostly reduced after catalytic reaction agree well with mechanistic studies performed on Cu-based catalysts in ethanol dehydrogenation: Cu^0^ and Cu^+^ have been shown as active species.^56^ In the case of the SEA catalyst, the originally amorphous structure changed, and the first hints of diffraction maxima appeared after catalysis in agreement with the STEM-EDS technique, which indicated particle growth. On the contrary, the width of diffractions in the DI sample remained very similar for fresh and spent catalysts. The Cu average coherent domain size, according to the Debye–Scherrer equation, reached ∼23 nm (compared with the Cu crystallite size of ∼32 nm in the fresh reduced catalyst). Noteworthily, this sample was the most stable during catalysis. HSG showed amorphous properties after the catalytic reaction corresponding to small nanoparticles. Finally, SHI exhibited weak but narrow diffraction, confirming the presence of some larger particles. Based on the XRD data and STEM-EDS analyses (where only small particles were observed), it can be concluded that the SHI catalyst experienced the most significant Cu migration and sintering. Notably, SHI also exhibited the lowest catalytic activity and the most significant deactivation.

## Conclusions

In this study, four Cu/SiO_2_ catalysts with different particle sizes were prepared to investigate the effect of particle size on catalytic activity and stability in non-oxidative ethanol dehydrogenation to acetaldehyde. The best Cu dispersion was observed in the case of the sample prepared by hydrolytic sol–gel (HSG). Small nanoparticles with narrow particle size distribution were prepared by strong electrostatic adsorption (SEA). Nanoparticles prepared by dry impregnation (DI) exhibited a larger deviation in size; both small and large particles were observed. The largest nanoparticles were prepared by the solvothermal hot-injection technique (SHI). Textural properties of SEA, DI, and SHI were similar to that of the silica support Aerosil 300 (interparticle porosity); only a slight decrease in SA_BET_ and *V*_total_ was observed for the samples upon copper introduction. The HSG sample exhibited different textural properties (intraparticle porosity). The reducibility of the catalysts was characterized by H_2_-TPR, and the temperature of the main peak followed the particle sizes estimated by STEM-EDS and XRD.

The HSG catalyst showed outstanding catalytic performance (65% ethanol conversion and >95% acetaldehyde selectivity at 255 °C). Thanks to the high WHSV used during the catalytic experiment, the HSG catalyst reached very high acetaldehyde productivity (2.79 g g^–1^ h^–1^; the second highest among the data reported under similar conditions and cited herein).^19^ The outstanding catalytic performance was assigned to high and homogeneous Cu dispersion that promotes the catalytic activity. However, the HSG catalyst was not stable; it suffered from coking and Cu particle sintering, similar to all other samples prepared within this study. The DI catalyst with larger particles and broader particle size distribution exhibited the most stable behavior during catalysis, the smallest coke deposition, and the highest nanoparticles stability against sintering (STEM-EDS and XRD) and maintained porosity without significant losses. This result is very important for potential renewable acetaldehyde production from ethanol, considering the very high acetaldehyde productivity (2.42 g g^–1^ h^–1^ at 255 °C) exhibited by DI and the ease of its preparation.

## Supporting Information Available

STEM-EDS micrographs (fresh, calcined samples), XPS spectra (Cu LMM region), N_2_ porosimetry results (Aerosil 300 support), XRD diffractograms (fresh calcined samples), and tables summarizing all catalytic data (PDF)

## Abbreviations

DI: dry impregnation
DFT: density functional theory
FID: flame ionization detector
GHSV: gas hourly space velocity
HAADF: high-angle annular dark-field
H_2_-TPR: temperature programmed reduction
HSG: hydrolytic sol–gel
ICP-OES: inductively coupled plasma optical emission spectroscopy
NPs: nanoparticles
ODE: octadecene
OLA: oleylamine
RFC: reactive frontal chromatography
SEA: strong electrostatic adsorption
SHI: solvothermal hot-injection synthesis
STEM-EDS: scanning transmission electron microscopy with electron dispersive spectroscopy
TEM: transmission electron microscopy
TGA: thermogravimetry analysis
UV–VIS: ultraviolet visible spectroscopy
WHSV: weight hourly space velocity
XPS: X-ray photoelectron spectroscopy
XRD: powder X-ray diffraction
