# Supplementary material for: Ethanol Dehydrogenation over Copper-Silica Catalysts: From Sub-Nanometer Clusters to 15 nm Large Particles
Source: ACS Sustain Chem Eng. 2023 Jul 20;11(30):10980–92. doi: 10.1021/acssuschemeng.2c06777 (PMC10394689; doi:10.1021/acssuschemeng.2c06777)
Supplement: Supplementary file 1 — sc2c06777_si_001.pdf [file sc2c06777_si_001.pdf]

Ethanol Dehydrogenation over Copper-Silica Catalysts: From Sub-Nanometer Clusters to 15 nm Large Particles

*Tomas Pokorny,[a] Vit Vykoukal,[a] Petr Machac,[a] Zdenek Moravec,[a] Nicola Scotti [b], Pavla Roupcova,[c,d] Katerina Karaskova,[e] Ales Styskalik[a]**

[a]Department of Chemistry, Masaryk University, Kotlarska 2, CZ-61137 Brno, Czech Republic

[b]CNR-SCITEC, Via Golgi 19, 20133 Milano, Italy.

[c]Institute of Physics of Materials, Academy of Sciences of the Czech Republic, Zizkova 22, CZ-616 62 Brno, Czech Republic

[d]CEITEC Brno University of Technology, Purkynova 123, 612 00 Brno, Czech Republic

[e]Institute of Environmental Technology, CEET, VSB-TUO, 17. listopadu 2172/15, 708 00 Ostrava, Czech Republic

*Corresponding author (styskalik@chemi.muni.cz).

Supporting information

Total number of pages: 10

Total number of figures: 7

Total number of tables: 3

Contents:

Figure S1. Properties of CuO nanoparticles synthesized by solvothermal hot injection used for deposition on silica support. pp. S3

Figure S2. Nitrogen adsorption and desorption isotherm of silica support Aerosil 300. pp. S4

Figure S3. STEM-EDS micrographs of fresh, calcined samples. pp. S5

Figure S4. Survey STEM-EDS micrographs of **DI** sample. pp. S6

Figure S5. XRD diffractograms of fresh, calcined samples. pp. S6

Figure S6. XPS analysis of Cu LMM region. pp. S7

Table S1. Ethanol conversion over different Cu-based catalysts. pp. S8

Table S2. Ethanol conversion during the catalytic stability tests. pp. S8

Table S3. Selectivity to acetaldehyde during the test of the catalytic activity pp. S9

Figure S7. STEM-EDS micrographs of spent Cu/SiO_2 catalysts. pp. S10

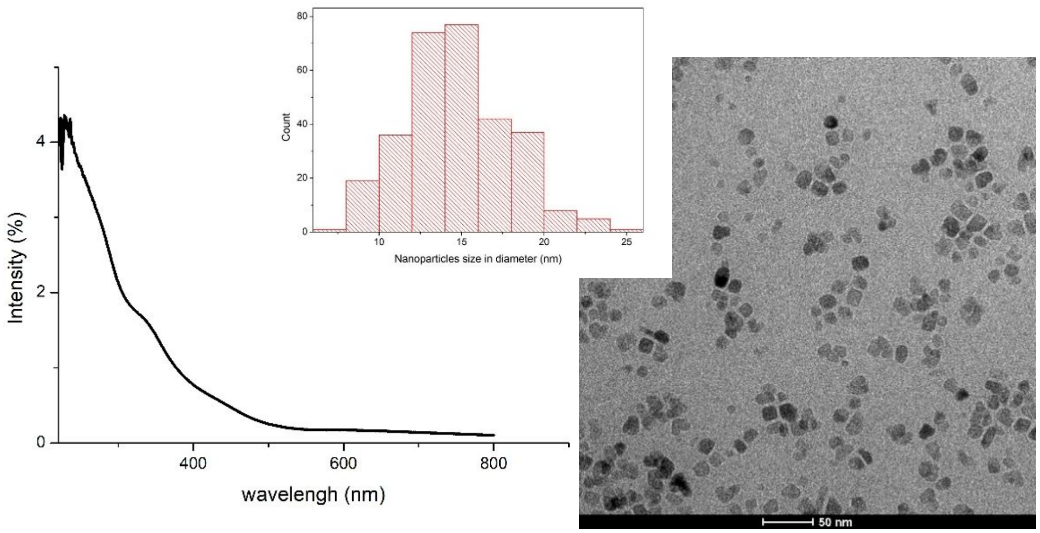

Figure S1. Properties of CuO nanoparticles synthesized by solvothermal hot injection used for deposition on silica support.

Nanoparticles synthesized by solvothermal hot injection were briefly characterized before their deposition on silica support (Figure S1). UV-Vis spectroscopy showed optical properties typical for CuO (without Cu plasmon resonance). The average size of these nanoparticles was 14.7 nm in their diameter, with a standard deviation of 3.1 nm.

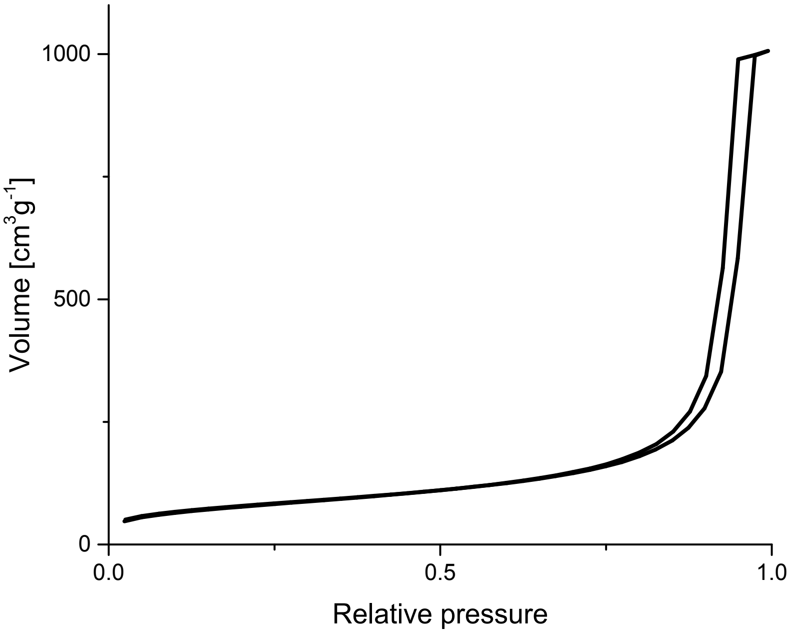

Figure S2. Nitrogen adsorption and desorption isotherm of silica support Aerosil 300 used for the deposition of copper. The surface area by BET reached 284 m^2 g^{-1} and pore volume 1.55 cm^3 g^{-1}.

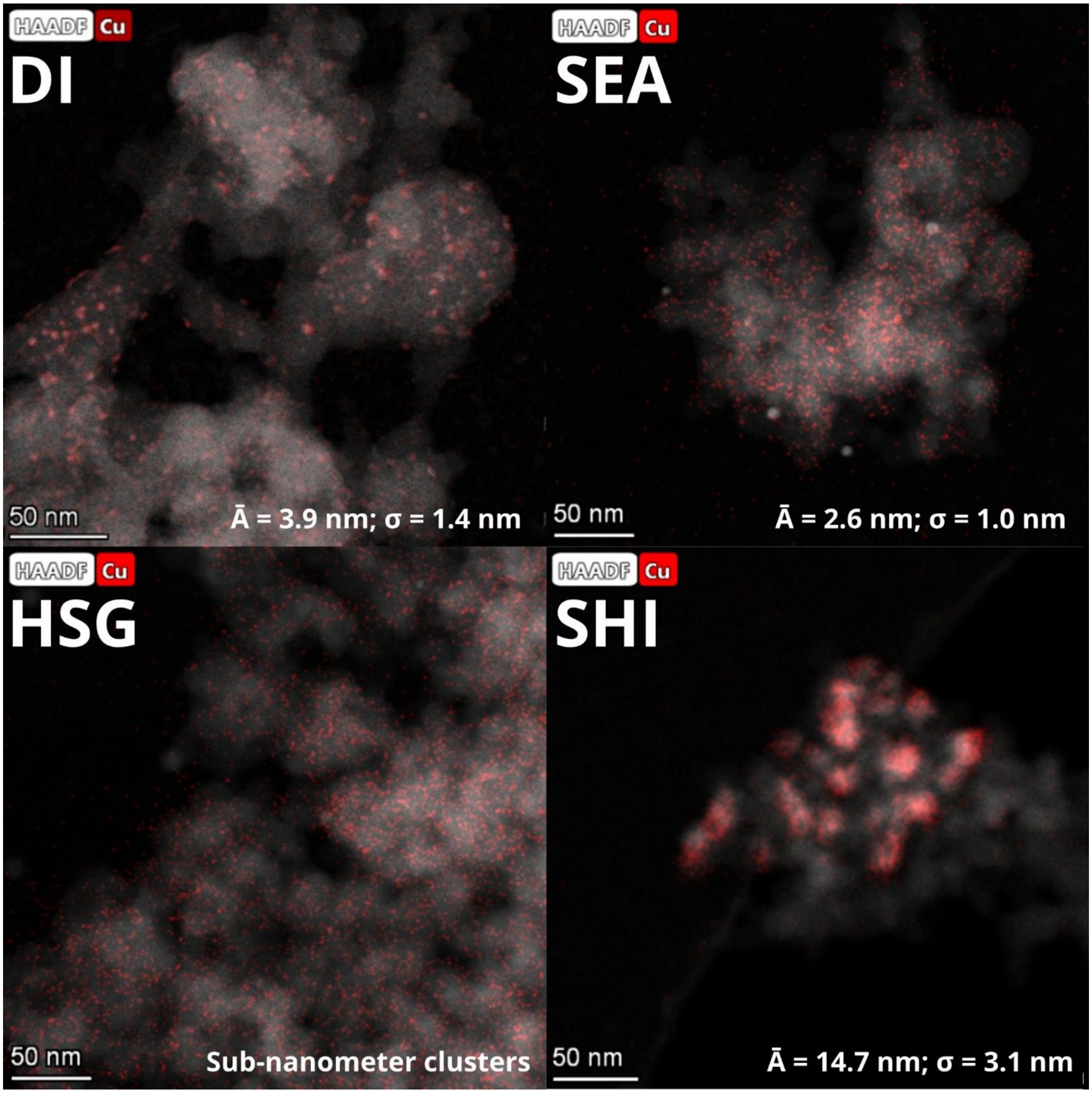

Figure S3. STEM-EDS micrographs of fresh, calcined samples.

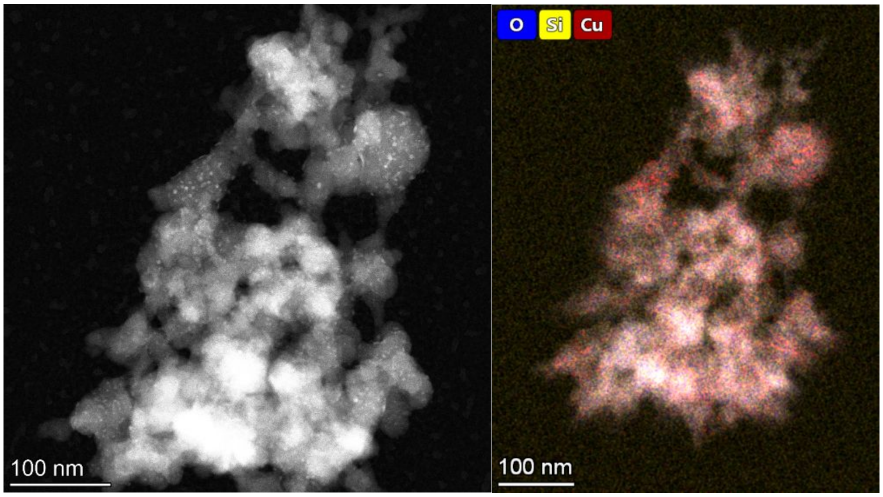

Figure S4. Survey STEM-EDS micrographs of **DI** sample.

Catalyst synthesized by dry impregnation (**DI**) provides a wide distribution of nanoparticles on the silica surface (up to 32 nm according to XRD). In this STEM-EDS micrograph (Figure S4) nanoparticles were analyzed by the ImageJ software, and they were in the range from 1.5 to 8 nm.

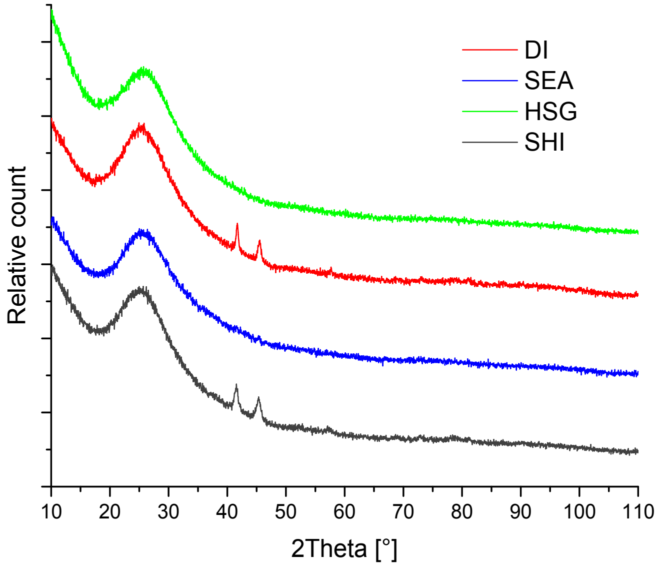

Figure S5. XRD diffractograms of fresh, calcined samples. The observed diffractions come from CuO (ICSD database No. 98-003-1059).

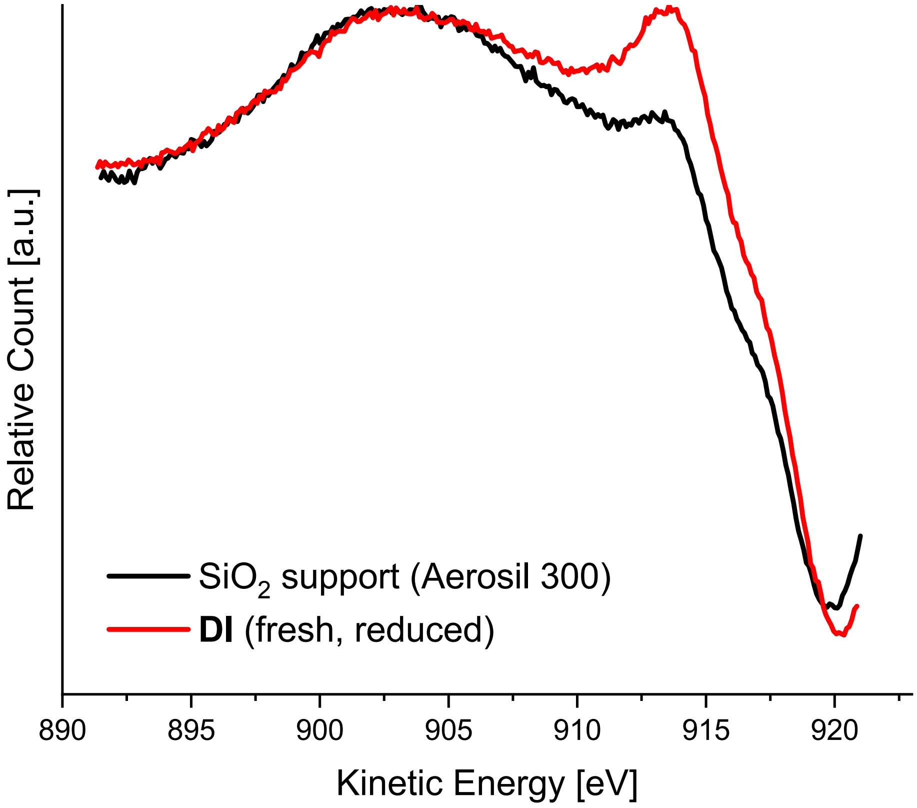

Figure S6. XPS analysis of Cu LMM region.

Table S1. Ethanol conversion (average of five measurements) over different Cu-based catalysts.

	Ethanol conversion at 185 ˚C [%]	Ethanol conversion at 220 ˚C [%]	Ethanol conversion at 255 ˚C [%]	Ethanol conversion at 290 ˚C [%]
DI	9	30	57	72
SEA	4	24	51	64
HSG	11	40	65	74
SHI	8	23	38	41

Table S2. Ethanol conversion [%] during the stability test of different Cu-based catalysts at 325 ˚C in ethanol dehydrogenation.

Time (hours)	1	2	3	4	5	6	7	8	9	10	11	12	13	14	15
DI	80	72	65	62	59	57	54	52	49	46	46	48	44	47	44
SEA	70	59	45	39	36	32	29	28	25	25	24	24	21	21	19
HSG	83	67	51	46	41	38	34	33	31	30	29	28	26	25	26
SHI	52	32	24	15	12	10	11	8	7	6	6	6	5	5	5

Table S3. Selectivity to acetaldehyde during the test of the catalytic activity.

	Selectivity to acetaldehyde 185 ˚C [%]	Selectivity to acetaldehyde 220 ˚C [%]	Selectivity to acetaldehyde 255 ˚C [%]	Selectivity to acetaldehyde 290 ˚C [%]
DI	84	91	94	96
SEA	98	95	96	97
HSG	88	92	95	97
SHI	45	75	87	89

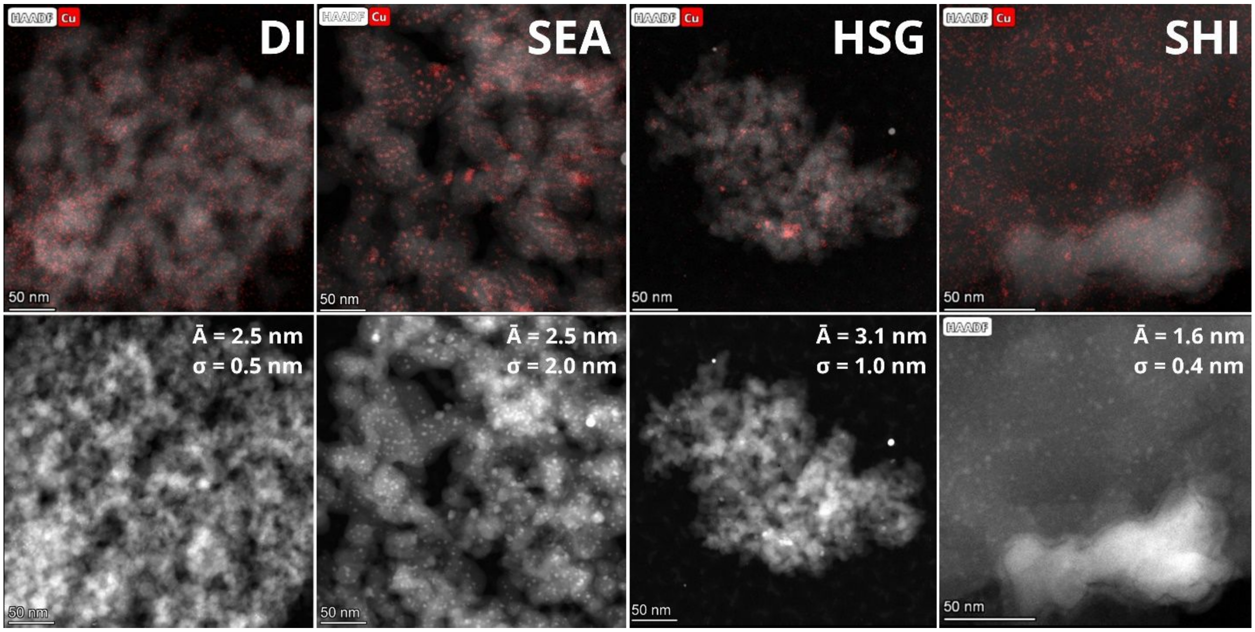

Figure S7. Comparison of STEM-EDS (top) and STEM with HAADF detector (down) micrographs of spent Cu/SiO_2 catalysts. Several bright spots in the micrographs of SEA and HSG catalysts are Au nanoparticles that contaminated samples during their preparation for STEM-EDS analysis.
